# Estimation of affinities of ligands in mixtures *via *magnetic recovery of target-ligand complexes and chromatographic analyses: chemometrics and an experimental model

**DOI:** 10.1186/1472-6750-11-44

**Published:** 2011-05-05

**Authors:** Xiaolan Yang, Yanling Xie, Jun Pu, Hua Zhao, Juan Liao, Yonghua Yuan, Sha Zhu, Gaobo Long, Chun Zhang, Huidong Yuan, Yiwen Chen, Fei Liao

**Affiliations:** 1 Unit for Analytical Probes and Protein Biotechnology, Key Laboratory of Medical Laboratory Diagnostics of the Ministry of Education, Department of Laboratory Medicine, Chongqing Medical University, Chongqing 400016, China; 2 College of Bioinformatics, Chongqing University of Post and Telecommunications, Chongqing 400065, China

## Abstract

**Abstract:**

**Background**

The combinatorial library strategy of using multiple candidate ligands in mixtures as library members is ideal in terms of cost and efficiency, but needs special screening methods to estimate the affinities of candidate ligands in such mixtures. Herein, a new method to screen candidate ligands present in unknown molar quantities in mixtures was investigated.

**Results**

The proposed method involves preparing a processed-mixture-for-screening (PMFS) with each mixture sample and an exogenous reference ligand, initiating competitive binding among ligands from the PMFS to a target immobilized on magnetic particles, recovering target-ligand complexes in equilibrium by magnetic force, extracting and concentrating bound ligands, and analyzing ligands in the PMFS and the concentrated extract by chromatography. The relative affinity of each candidate ligand to its reference ligand is estimated *via *an approximation equation assuming (a) the candidate ligand and its reference ligand bind to the same site(s) on the target, (b) their chromatographic peak areas are over five times their intercepts of linear response but within their linear ranges, (c) their binding ratios are below 10%. These prerequisites are met by optimizing primarily the quantity of the target used and the PMFS composition ratio.

The new method was tested using the competitive binding of biotin derivatives from mixtures to streptavidin immobilized on magnetic particles as a model. Each mixture sample containing a limited number of candidate biotin derivatives with moderate differences in their molar quantities were prepared *via *parallel-combinatorial-synthesis (PCS) without purification, or *via *the pooling of individual compounds. Some purified biotin derivatives were used as reference ligands. This method showed resistance to variations in chromatographic quantification sensitivity and concentration ratios; optimized conditions to validate the approximation equation could be applied to different mixture samples. Relative affinities of candidate biotin derivatives with unknown molar quantities in each mixture sample were consistent with those estimated by a homogenous method using their purified counterparts as samples.

**Conclusions:**

This new method is robust and effective for each mixture possessing a limited number of candidate ligands whose molar quantities have moderate differences, and its integration with PCS has promise to routinely practice the mixture-based library strategy.

## Background

Ligands of proteins are widely used as clinical drugs, and as research and analytical tools. To discover valuable ligands that possess strong affinities to a desired binding site on a protein target, the combinatorial library strategy is a powerful method, but it is usually thwarted by the cost and time involved in preparing and screening library members[1-9]. In theory, an ideal approach to the combinatorial library strategy would integrate the preparation of multiple candidate ligands in each mixture as a library member with the simultaneous estimation of affinities and the concurrent judgment of binding site(s) of all candidate ligands in the mixture as a sample. This integration approach belongs to the mixture-based library strategy, and may have pronounced advantages to discover valuable optically-active ligands because it is easy to prepare racemic reaction mixtures instead of optically-pure enantiomers as library members[3,4,6,9].

Nowadays, to practice the mixture-based library strategy, mixtures of multiple candidate ligands are routinely prepared *via *the pooling of individual compounds, parallel combinatorial synthesis (PCS) or the fractionation of crude extracts of natural products. The preparation of mixtures of multiple candidate ligands *via *the pooling of individual compounds suffers from the cost and time required for synthesizing individual compounds, but the composition ratios of candidate ligands in these mixtures can be rigorously controlled. The preparation of mixtures of multiple candidate ligands *via *PCS or the fractionation of crude extracts of natural products has attractive cost and efficiency. However, the composition ratios of candidate ligands in these mixtures cannot be rigorously controlled when PCS is utilized, while such composition ratios can hardly be controlled at all when the fractionation of crude extracts of natural products is used.

Currently, high-throughput-screening techniques are commonly used to quantify affinities of candidate ligands from libraries, but they require individual compounds with purities of over 80%, rather than mixtures of compounds, as library members[1,2,5,8,9]. To concurrently estimate the affinities of multiple candidate ligands in the aforementioned mixtures, several screening methods are available as discussed below; however, they all suffer some disadvantages. (a) The frontal-affinity-chromatography technique has ideal efficiency, but requires affinity columns of short life-times and exhibits low reliability to judge the binding site(s) of multiple candidate ligands of unknown concentration in a mixture under analysis[10,11]. (b) The size-exclusion-chromatography affinity-selection mass-spectrometry technique measures the affinities of multiple candidate ligands in a mixture, but requires special instrumentation and is inapplicable to multiple candidate ligands of unknown concentration in a mixture[11-15]. (c) High-performance-liquid-chromatography mass-spectrometry (HPLC-MS) coupled to ultra-filtration determines the affinities of multiple candidate ligands of unknown concentration in a mixture, but requires protein targets of high thermostability and exhibits too low efficiency[13,16,17]. (d) Indirect deconvolution techniques show favorable efficiency in the screening of thousands of candidate ligands of unknown concentration in a mixture, but rely on synthetic reactions of rigorous yields and accurate assays of activities of ligand mixtures[18-21]. (e) Direct deconvolution techniques are very efficient for screening multiple candidate ligands of unknown concentration in a mixture, but the design, preparation and analysis of the chemical tags for coding candidate ligands in each mixture is quite challenging[18-21]. (f) The diffusion-based method measures the affinities of multiple candidate ligands in mixtures, but is applicable mainly to candidate ligands of known concentration in a mixture[22]. Hence, new methods are needed to screen multiple candidate ligands of unknown concentration in mixture samples.

In general, to simultaneously quantify affinities of multiple candidate ligands in a mixture, both the separation of the target-ligand complexes from free ligands and chromatographic analyses of ligand mixtures are required. It is known that target-ligand complexes can be readily recovered *via *magnetic force with satisfactory recovery ratios and practical efficiency as long as protein targets are immobilized on magnetic particles[23-29]. Thus far, however, the magnetic recovery of target-ligand complexes coupled to chromatographic analyses of ligand mixtures has failed to quantify affinity(ies) of candidate ligand(s) in mixtures[26-29]. Therefore, based on the magnetic recovery of target-ligand complexes and chromatographic analyses of ligand mixtures, we report here a new method to estimate the affinities of a limited number of candidate ligands possessing moderate differences in their molar quantities in each mixture as a sample. Chemometrics to approximate ligand affinities and strategies to optimize principal factors are proposed, and their effectiveness has been tested with competitive binding of biotin derivatives in mixtures to streptavidin immobilized on magnetic nanoparticles (SMPP) as a model.

## Results

### Chemometrics to estimate affinities of candidate ligands in a mixture

**Figure 1:**
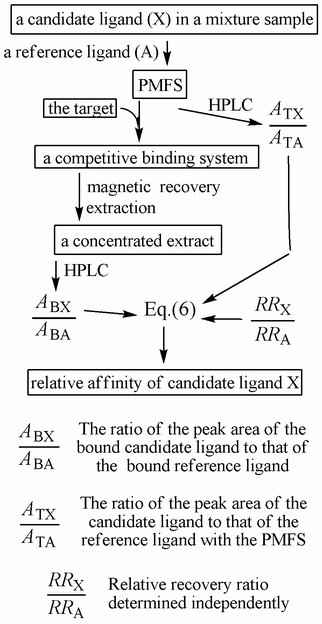
**Basic operation steps to realize this new method**.

By employing an exogenous reference ligand to each mixture sample, this new method utilizes the basic steps shown in Figure 1, and is distinctively characterized by the preparation of a processed-mixture-for-screening (PMFS) with the mixture sample and the reference ligand. In practice, such a PMFS and the target immobilized on magnetic particles are mixed to initiate competitive binding; target-ligand complexes in equilibrium are recovered *via *magnetic force; bound ligands are extracted with suitable solvent(s) and concentrated according to a preset concentration ratio. Then, candidate ligand(s) and the reference ligand in both the PMFS and its concentrated extract are quantified using a chromatographic system under identical conditions (the volumes of solutions loaded for chromatographic analyses are kept constant). Finally, the relative affinity of each candidate ligand, which is quantifiable together with the reference ligand in the PMFS and its concentrated extract, is approximated *via *Eq.(6) as described below.

To derive Eq.(6), the following parameters are defined for a pair of a candidate ligand and its reference ligand.

*A*_TA_: peak area for the reference ligand in a competitive binding system, which is determined directly with the PMFS after proper dilution;

*A*_TX_: peak area for a quantifiable candidate ligand in a competitive binding system, which is determined directly with the PMFS after proper dilution;

*A*_BA_: peak area for the bound reference ligand in the concentrated extract from the competitive binding system;

*A*_BX_: peak area for the bound candidate ligand in the concentrated extract from the competitive binding system;

*N*_FS_: the quantity of total unoccupied binding site(s) on the protein target used in the competitive binding system;

*CR*: the ratio of the volume of the competitive binding system to the volume of the concentrated extract of bound ligands to be analyzed by a chromatographic system;

*K*_A_: the equilibrium constant for the binding of the reference ligand to the target;

*K*_X_: the equilibrium constant for the binding of the candidate ligand to the target;

*RR*_A_: the overall recovery ratio of the reference ligand after the whole operation process;

*RR*_X_: the overall recovery ratio of the candidate ligand after the whole operation process;

*BR*_A_: the binding ratio of the reference ligand in the competitive binding system;

*BR*_X_: the binding ratio of the candidate ligand in the competitive binding system;

The reference ligand and the candidate ligands, which are simultaneously quantifiable using a chromatographic system, are referred to as ligands of interest hereafter. The linear response of chromatographic peak areas of any resolved component to its quantities loaded for analyses yields a slope, an intercept and a linear range. If each ligand of interest, in both the PMFS and the concentrated extract, produces peak areas within the linear range but over five times the absolute value of the intercept of linear response, the intercept can be neglected to derive the quantities of the ligand of interest under analyses from the peak areas. In this case, Eq.(1) and Eq.(2) approximate the binding equilibrium constants for the reference ligand and the candidate ligand, respectively, while Eq.(3) and Eq.(4) approximate their corresponding binding ratios.(1)(2)(3)(4)

The relative affinity of the candidate ligand to the reference ligand is defined as *K*x/*K*A. In Eq.(1) and Eq.(2), *CR *is the same for the reference ligand and the candidate ligand because they co-exist in both the same concentrated extract of bound ligands and the same competitive binding system. If the candidate ligand and the reference ligand in the PMFS bind to the same site(s) on the target, *N*_FS _is the same in Eq.(1) and Eq.(2), and thus Eq.(5) applies.(5)

*RR*A and *RR*x are determined concurrently with a PMFS or any similar mixture of the reference ligand and the candidate ligand. When the binding ratios of the reference ligand and the candidate ligand are below 10%, *A*BA/(*RR*A ×*CR*) and *A*BX/(*RR*x/*CR*) are negligible in comparison to *A*_TA _and *A*_TX_[30], respectively, and thus Eq.(6) applies.(6)

Clearly, the reliability of the relative affinity of a candidate ligand relies on the validity of Eq.(6), and requires the simultaneous satisfaction of the following prerequisites for the pair of the candidate ligand and its reference ligand. (a) The candidate ligand and its reference ligand bind to the same site(s) on the target. In practice, the binding site(s) of the candidate ligand can be judged based on its competitive binding against a reference ligand but can not be optimized. (b) The candidate ligand and its reference ligand, in both the PMFS and the concentrated extract, produce peak areas within their own linear ranges. (c) The candidate ligand and its reference ligand, in both the PMFS and the concentrated extract, produce peak areas over five times the absolute values of their own intercepts of linear response. (d) The candidate ligand and its reference ligand have binding ratios of below 10% in the competitive binding system. All the later three prerequisites should be met by optimizing the experimental conditions.

For a pair of a candidate ligand and its reference ligand in both the PMFS and the concentrated extract, the satisfaction of the prerequisites of Eq.(6) for their peak areas should be judged before assessing the satisfaction of the prerequisites for their binding ratios. For each ligand of interest, the intercept and the range of linear response for assessing the satisfaction of prerequisites of Eq.(6) can be indexed by the peak areas, and are usually determined with the purified counterpart, but such a purified counterpart is unavailable with a mixture-based library. In practice, however, the intercept and the linear range indexed by peak areas for each ligand of interest can be estimated from the response of its peak areas to the quantities of the PMFS; such parameters should be consistent with those obtained using a purified counterpart, and can be used to judge the satisfaction of the prerequisites of Eq.(6).

To use this new method for candidate ligand(s) of unknown quantity in a mixture sample, the quantity of the target used and the composition ratio of all ligands of interest in a PMFS of the mixture sample are the principal factors to be optimized. A parameter-dependent or experimental approach can be used to estimate an optimized quantity of the target, while a rough or fine optimization way can be used to establish an optimized PMFS composition ratio.

For estimating an optimized quantity of target, the parameter-dependent approach requires some common and individual parameters. The common parameters of different mixture samples include the volumes of solutions loaded for chromatographic analyses, the concentration ratio and the binding capacity of the target. The individual parameters for each ligand of interest in a PMFS include its recovery ratio, its slope and intercept of linear response by chromatographic analyses. The slope of linear response of a candidate ligand can be estimated from the response of its peak areas to the quantities of the PMFS, and the content of the candidate ligand in its mixture sample prepared *via *PCS can be approximated with information from databases such as CASREACT http://www.cas.org/expertise/cascontent/casreact.html. From such parameters, the minimum quantity of target is calculated for a ligand of interest in a concentrated extract to produce a peak area that meets the prerequisites of Eq.(6) (additional file 1). The sum of such minima of target required for all ligands of the same binding site from a PMFS is an optimized quantity of the target. In general, any practical quantity of target that can be applied to different mixture samples is more desirable, but it cannot be reliably approximated due to the propagation of errors and competitive binding among ligands from any PMFS.

For estimating an optimized quantity of target, the experimental approach determines the response of peak areas of each ligand of interest in the concentrated extracts from a PMFS of a mixture sample to the quantities of target used. From this response, a minimum quantity of target for the peak area of each ligand of interest in a concentrated extract to meet the prerequisites of Eq.(6) is predicted. The maximum among such minima of target for all ligands of interest from the PMFS is an optimized quantity of target for the mixture sample. A practical quantity of target that is reasonably higher than such an optimized one estimated with a representative mixture sample is expected to be applied to different mixture samples.

The fine optimization way to establish an optimized PMFS composition ratio involves the testing of a series of the PMFSs whose concentrations of ligand(s) of interest are increased stepwise. For any quantifiable candidate ligand, the response of the relative affinities, which are calculated *via *Eq.(6) without judging its validity, to PMFS composition ratios is checked. Usually, the quantities of the mixture sample in PMFSs are increased stepwise from a low initial value while the molar quantity of the reference ligand in competitive binding systems is fixed at a value where the binding ratio of the reference ligand is about 10% in the absence of any other ligand. For a candidate ligand whose affinity is much greater than that of the reference ligand, the quantities of the reference ligand in the PMFSs should be increased stepwise from a low initial value while the quantity of the mixture sample in the PMFSs is fixed. The reliable relative affinity of any candidate ligand is independent of its concentrations in competitive binding systems. Thus, during the stepwise adjustment of PMFS composition ratios from a low initial value, relative affinities of any quantifiable candidate ligand calculated *via *Eq.(6) without judging its validity will become stable after Eq.(6) is finally validated. The average, or any one, of the stable relative affinities indexes the relative affinity of the candidate ligand, and any PMFS composition ratio to give such a relative affinity of the candidate ligand is an optimized one. An optimized PMFS composition ratio for a candidate ligand may not necessarily apply to another candidate ligand in the same mixture sample, and thus it is hard to establish an optimized PMFS composition ratio that can be applied to different mixture samples.

The rough optimization way to establish an optimized PMFS composition ratio involves testing of just a few PMFSs, in which the concentrations of ligand(s) of interest are increased exponentially. With each PMFS, the validity of Eq.(6) is judged for each pair of a quantifiable candidate ligand and its reference ligand before the preparation of the consecutive PMFS. The concentration of a candidate ligand in its mixture sample, and parameters of linear response of ligands of interest can be approximated as described just above. In practice, in the first PMFS, the quantity of the mixture sample is preset for binding ratio(s) of about 10% for (most) candidate ligand(s) in the absence of the reference ligand while the concentration of the reference ligand is preset at a value where its binding ratio is about 10% in the absence of any candidate ligand. If Eq.(6) is already validated for a pair of a candidate ligand and the reference ligand with the first PMFS, the relative affinity of the candidate ligand is available and the composition ratio of the first PMFS is an optimized one for the candidate ligand. Otherwise, the contents of the mixture sample (or the concentrations of the reference ligand) in the consecutive PMFSs are increased exponentially until Eq.(6) is validated for the pair of the candidate ligand and the reference ligand (additional file 2). In general, the molar quantity of all ligands of interest from the first PMFS should be considerably large in the competitive binding systems to validate Eq.(6) simultaneously for as many candidate ligands as possible in the first PMFS, and the optimized composition ratio of the first PMFS from a representative mixture sample can be applied to different mixture samples.

The sequential optimizations of experimental conditions till Eq.(6) is validated for each pair of a quantifiable candidate ligand from a mixture sample and a suitable reference ligand will ultimately give the relative affinity of every quantifiable candidate ligand in the mixture sample. The comparison of the relative affinities of all candidate ligands from a library provides information on valuable ligands. A candidate ligand bearing high affinity to the desired binding site(s) on the target is a hit from the library.

### Design of experimental models

Experimental conditions can be easily optimized to validate Eq.(6) for just one pair of a quantifiable candidate ligand from a mixture sample and its reference ligand. Therefore, this new method was firstly tested with each mixture sample containing a single candidate ligand as the primary component (the affinities of other compounds as the secondary components are not estimated), and then tested with each mixture sample containing multiple candidate ligands.

To detect all biotin derivatives in a series of mixture samples by HPLC-mass-spectrometry (HPLC-MS) with just one exogenous reference ligand, some biotinyl derivatives producing positive ion signals upon electro-spray-ionization (ESI) were designed, including *N*-biotinyl-benzylamine (BBZA), *N*-biotinyl-*N*'-(1-naphthyl)-ethylenediamine (BNEDA), methyl biotin ester (BME), *N*-(*N*_α_-biotinyl-phenylalaninyl)-*N*'-dansyl-ethylenediamine (BPDEDA), *N*-biotinyl-*N*'-dansyl-ethylenediamine (BDEDA), *N*-biotinyl-cyclohexylamine (BCHA), *N*-biotinyl-diethanolamine (BDETA) and *N*-biotinyl-morpholine (BMPL)[31,32] (additional file 3 and additional file 4). Natural biotin which had a negligible signal in the positive ion mode was used as the reference ligand to judge the binding site(s) of candidate ligands.

To prepare a single candidate biotin derivative in each mixture *via *PCS without purification as a sample, BCHA (cyclohexylamine is a primary alkyl amine), BDETA (diethanolamine is a secondary alkyl amine), BME (methanol is an alkyl alcohol) were used as potential specific candidate ligands, respectively, as large differences in their yields were expected after limited reaction periods with *N*-hydroxylsucciniimide biotin ester (NHS-Biotin)[31,32].

**Figure 2:**
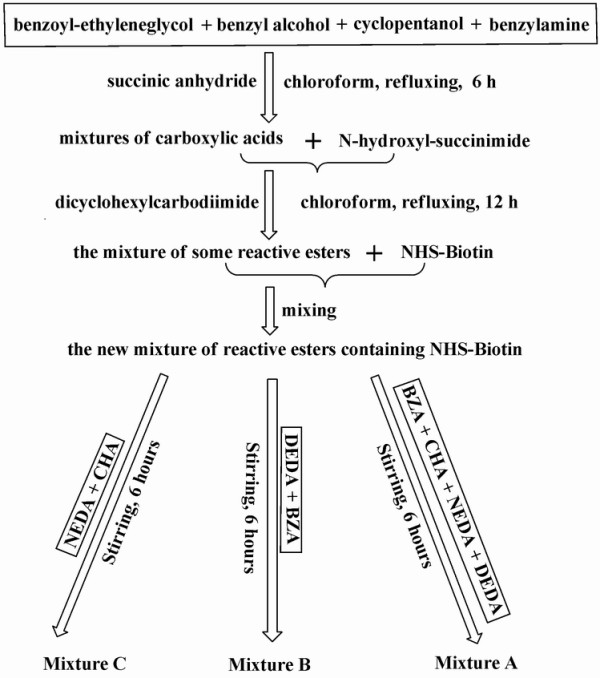
**SPSAG to prepare mixture samples of multiple biotin derivatives**.

To prepare multiple candidate ligands in mixture samples, BCHA, BBZA, BNEDA and BDEDA were used as potential specific candidate ligands. (1) To prepare mixtures *via *the pooling of individual compounds, *N*, *N*'-didansyl-ethylenediamine (DDEDA) and di-(4-*N*-acetylaniline) methane (DNAM) were used as the expected nonspecific candidate ligands of negligible affinities, and they were pooled with potential specific candidate biotin derivatives. (2) To prepare mixtures *via *PCS, solution-phase simultaneous addition of groups (SPSAG) was used[33]. In brief, a mixture of some *N*-hydroxylsuccinamide esters including NHS-Biotin was added to each mixture of some primary alkyl amines in slight excess (Figure 2)[31,32]. Comparable reaction rates of primary alkyl amines with NHS-Biotin support the postulation that the molar quantity of each candidate biotin derivative in the reaction mixture is determined by the molar quantity of the corresponding alkyl primary amine. In these mixtures *via *SPSAG, all the non-biotin components with positive ion signals upon ESI were potential nonspecific candidate ligands (additional file 5). (3) To test the universal applicability of the optimized conditions, three mixture subtypes were prepared as defined below. (a) Mixture A contained the four (expected) candidate biotin derivatives (BCHA, BBZA, BNEDA and BDEDA) with (expected) equal molar ratio; (b) Mixture B contained (expected) BDEDA and BBZA at the (expected) molar ratios of 1:3 and 3:1; (c) Mixture C contained (expected) BNEDA and BCHA at the (expected) molar ratios of 1:3 and 3:1.

### Estimation of the affinity of a single candidate biotin derivative in a mixture

#### HPLC-MS analyses of mixtures

**Figure 3:**
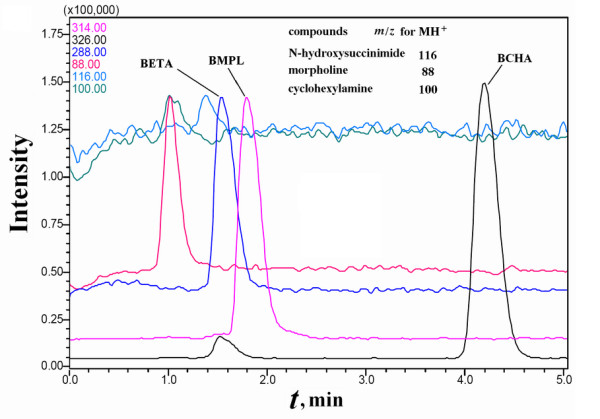
**HPLC-MS-SIM analyses of a PMFS of BCHA alone as the ligand of interest**. The PMFS of BCHA was prepared with purified BMPL plus morpholine. N-biotinyl ethanolamine (BETA) was detected as a secondary ligand.

**Figure 4:**
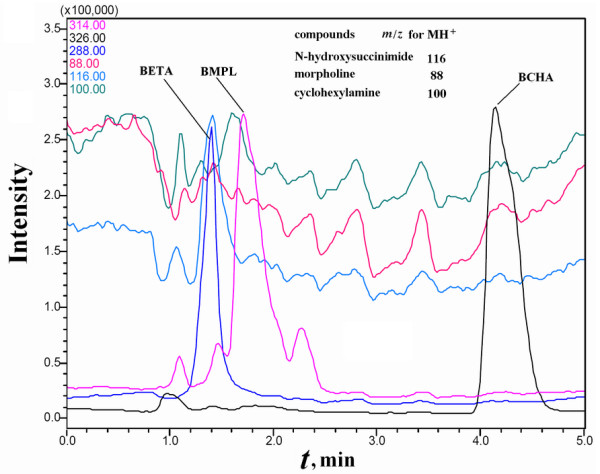
**HPLC-MS-SIM analyses of the concentrated extractof bound ligands from the PMFS of BCHA alone as the ligand of interest**. The PMFS was that used in Figure 3. The component in the concentrated extract with *m*/*z *= 116 and a retention time of 1.4 min was a contaminant from SMPP.

Purified BMPL was used as the reference ligand for a single candidate biotin derivative in each mixture sample. By using HPLC-MS *via *selective ion monitoring (HPLC-MS-SIM) for M+H^+ ^of the biotin derivatives (BMPL plus any one of BCHA, BDETA and BME), some contaminants originating from SMPP with the same *m*/*z *as the biotin derivatives were detected, but the biotin derivatives of interest in any PMFS and those in the concentrated extract could be readily resolved from the contaminants (Figure 3 and 4). The use of ethanolamine to remove residual NHS-Biotin produced *N*-biotinyl-ethanolamine (*m*/*z *= 288 for M+H^+^) in the designed mixture samples as a secondary component whose affinity was not estimated.

The peak areas of each purified candidate biotin derivative or BMPL responded linearly to the quantities loaded for HPLC-MS-SIM analyses (Table 1, and additional file 6). The upper limit of the linear response for any tested biotin derivative reached 30 pmol. BCHA had the largest slope of linear response while BME gave the smallest slope of linear response; the difference in their slopes was 17-fold. The intercepts of linear response for these purified biotin derivatives were all greater than zero and had differences comparable to those in their slopes.

**Table 1:** Parameters for HPLC-MS-SIM analyses and approximation of the minimum quantity of SMPP with each mixture sample of a single candidate biotin derivative.

Compound	BCHA	BMPL	BDETA	BME
*m/z *for M+H^+^	326	314	332	259

Slope for linear response^a^	862.1	279.8	116.9	49.2
Intercept for linear response^a^	322.1	143.4	59.9	24.2
*R*^2 ^for linear response	> 0.998	> 0.998	>0.994	>0.992
Minimum quantity of the ligand in 5 μL solution to meet the prerequisites of Eq.(6) (pmol)^b^	1.5	2.0	2.1	2.0
Minimum quantity of the ligand in 40 μL extract to meet the prerequisites of Eq.(6) (pmol)	12	16	16.8	16
Recovery ratio^c^	0.47	1.00	0.57	0.82
Minimum quantity of the ligand bound to SMPP in competitive binding systems (pmol)^d^	26	16	31.5	20
Minimal quantity of SMPP to bind the ligand (μL)^e^	6.5	4.0	7.8	5.0
Summed minima of SMPP for a PMFS (μL)	11	-	12	9

^a ^both peak area and intercept were in μA.ms and the quantity of each pure biotin derivative was in pmol.

^b ^calculated as the quantity of the biotin derivative for peak areas five times of the absolute value of its intercept for linear response.

^c ^determined in triplicate with variation coefficient below 9%.

^d ^calculated considering recovery ratio to give the required extract of bound ligand.

^e ^calculated with the binding capacity of 4 nmol per mL SMPP for a extract of equal contents of one candidate ligand and BMPL as the reference ligand.

When calibrated with a pure counterpart, the mass content of each candidate biotin derivative in its reaction mixture prepared *via *PCS was over 50%, with the highest content being over 70% for BDETA and the lowest content being about 55% for BCHA. The peak areas of each candidate biotin derivative and BMPL in a PMFS also responded linearly to the quantities of the PMFS loaded for HPLC-MS-SIM analyses. The difference in the intercepts of linear response for any purified candidate biotin derivative and its counterpart in PMFS was below 20%, whereas the difference in the linear ranges was negligible. However, the difference in slopes for the purified BDETA and its counterpart in PMFS was about 35%, which was the largest value found.

#### Optimization of the quantity of SMPP and the concentration ratio

The recovery ratio of each candidate biotin derivative (BCHA, BDETA or BME), as well as that of BMPL, was over 40% with a small coefficient of variation (CV), and the relative recovery ratio of each candidate biotin derivative to BMPL showed an even smaller CV (Table 1). The highest sensitivity to quantify BCHA facilitated its use to titrate the binding sites on SMPP, which was about 4.2 nmol BCHA per mL SMPP. When 50 μL SMPP was used in competitive binding systems and the extracts of bound ligands were concentrated into 40 μL methanol, HPLC-MS-SIM analyses of each biotin derivative of interest (BCHA, BDETA, BME or BMPL) in concentrated extracts confronted with negligible interference of contaminants from SMPP. When such extracts were concentrated into 8 μL, however, some contaminants with the same *m*/*z *caused interference with HPLC-MS-SIM analyses of the biotin derivatives of interest.

For establishing an optimized quantity of SMPP by the parameter-dependent approach, the parameters of linear response for each purified biotin derivative were used. The minimum quantity of SMPP in a competitive binding system was estimated for each biotin derivative in 40 μL extract to produce a peak area over five times the intercept of linear response. The sum of such SMPP minima with a PMFS of each mixture sample was consistently about 10 μL (Table 1, and additional file 1). For the universal applicability to all designed mixture samples and the consideration of the potential binding of *N*-biotinyl-ethanolamine, SMPP at 50 μL was used in each competitive binding system of 2.0 mL and each extract was concentrated into 40 μL methanol. This practical quantity of SMPP was about four times the sum of the SMPP minima estimated *via *the parameter-dependent approach using linear responses by HPLC-MS-SIM analyses of purified biotin derivatives, but was over seven times the sum of the SMPP minima estimated from parameters of linear response with PMFSs (data not given).

#### Optimization of PMFS composition ratios to estimate ligand affinity

**Figure 5:**
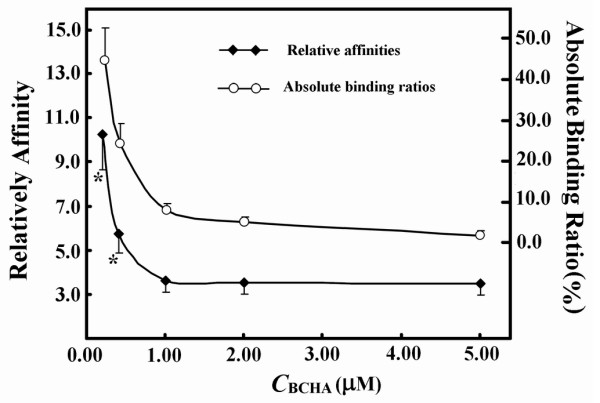
**Response curves of relative affinities and binding ratios of BCHA to its concentrations in the competitive binding systems containing 1.0 μM BMPL**. *indicated significant difference from the stable values after Eq.(6) was validated. Data were from assays in duplicate with CVs of below 12%.

**Figure 6:**
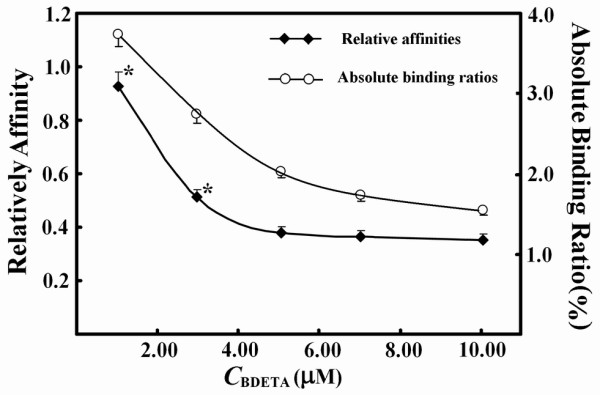
**Response curves of relative affinities and binding ratios of BDETA to its concentrations in the competitive binding systems containing 1.0 μM BMPL**. *indicated significant difference from the stable value after Eq.(6) was validated. Data were from assays in duplicate with CVs of below 10%.

The fine optimization way was tested to establish an optimized PMFS composition ratio. BMPL as the reference ligand in the competitive binding systems was fixed at 1.0 μM for its binding ratio of about 10% in the absence of other ligands. As calculated *via *Eq.(6) without judging its validity, the relative affinities of each candidate biotin derivative (BCHA, BDETA, or BME) gradually became stable during the stepwise adjustment of PMFS composition ratios. (a) BCHA and BME have higher affinities than BMPL. The peak areas of BCHA or BME in concentrated extracts were usually five times higher than the absolute value of the intercept of linear response. However, when the concentrations of BCHA in its PMFSs were low, its binding ratios usually exceeded 10%, and its relative affinities calculated *via *Eq.(6) showed large positive deviations from those with BCHA at higher concentrations in its PMFSs. The stepwise increase in the concentrations of BCHA in the PMFSs led to a continuous decrease in the binding ratios, and its relative affinities calculated *via *Eq.(6) gradually decreased to stable values after the binding ratios of BCHA were below 10% (Figure 5). After this point, any further increase in its concentrations in PMFSs reduced the binding ratios but exerted few effects on its relative affinities. Similar results were observed with BME. (b) BDETA has a lower affinity than BMPL. The binding ratios of BDETA were always below 10% while its peak areas with concentrated extracts usually invalidated Eq.(6), when its concentrations in PMFSs were relatively low. Similarly, the stepwise increase in BDETA concentrations in PMFSs led to continuous decrease in its binding ratios and its relative affinities finally decreased to stable values after its peak areas with concentrated extracts were over five times its intercept of linear response (Figure 6). (c) The average, or any one, of the stable relative affinities of each candidate biotin derivative (BCHA, BDETA, or BME) on the response curve was taken as the index of its relative affinity, and this relative affinity was consistent with that obtained using a homogenous method and its purified counterpart as the sample (Table 2)[32].

**Table 2:** Relative affinity of a single candidate biotin derivative in its mixture sample prepared *via *PCS.

compounds	Relative affinity	Relatively affinity *via *Eq.(6)	
	Homogenous method	Fine optimization^a^	Rough optimization^b^
BMPL	1.00	1.00	
BCHA	3.68 ± 0.54 (5)	3.13 ± 0.27 (6)	3.23 ± 0.12 (2)
BME	1.90 ± 0.34 (2)	1.85 ± 0.25 (6)	1.65 ± 0.15 (2)
BDETA	0.45 ± 0.06 (4)	0.41 ± 0.11 (6)	0.51 ± 0.05 (2)

Number in parenthesis was for independent assays. For each candidate biotin derivative, different methods yielded consistent relative affinities.

^a ^The average of the stable relative affinities on the response curve.

^b ^The result was from the second PMFS containing the approximated candidate biotin derivative at 5.0 μM.

The rough optimization way to establish an optimized PMFS composition ratio was also tested. When the concentration of BME (or BDETA) from its mixture sample was equal to that of BMPL as the reference ligand in the first PMFS, Eq.(6) was validated to give the relative affinity that was consistent with that obtained by the fine optimization way. However, when the concentration of BCHA from its mixture sample was equal to that of BMPL in the first PMFS, the prerequisites for a validated Eq.(6) with BCHA and BMPL were not met simultaneously. After the concentration of BCHA from the mixture sample in the second PMFS was increased by four-fold, all the prerequisites to validate Eq.(6) were met concurrently, and the relative affinity of BCHA was consistent with that obtained by the fine optimization way (Table 2).

### Estimation of affinities of multiple candidate biotin derivatives in a mixture

#### HPLC-MS analyses of mixtures

BPDEDA was used as the exogenous reference ligand for multiple candidate biotin derivatives in each mixture sample. By HPLC-MS-SIM analysis of a PMFS from Mixture A *via *the pooling of individual compounds, the five biotin derivatives (BCHA, BBZA, BNEDA, BDEDA and BPDEDA at equal concentrations) and other detected components were readily resolved from each other. Peak areas of each detected component in this PMFS responded linearly to the quantities of the PMFS loaded for HPLC-MS-SIM analyses (additional file 7). The intercept of linear response for BBZA was below zero while those of the other biotin derivatives were over zero. The slope of linear response for BCHA was comparable to that for BDEDA and was higher than those for other biotin derivatives, but the slope of linear response for BNEDA was the lowest among those for the five biotin derivatives.

#### Optimization of the quantity of the target and PMFS composition ratios

In the 40 μL concentrated extract from a competitive binding system containing 0.20 mL SMPP and the PMFS of Mixture A *via *the pooling of individual compounds, each bound biotin derivative was resolved from other components (additional file 8). The recovery ratios of DNAM and DDEDA were over 35% while those of the five biotin derivatives in the PMFS of Mixture A were consistently over 40%.

The parameter-dependent approach was tried at first to estimate an optimized quantity of SMPP using linear responses from HPLC-MS-SIM analyses of a PMFS of Mixture A, which served as the representative mixture because its number of candidate ligands was the largest. The sum of the minimum quantities of SMPP was about 13 μL, for all the five biotin derivatives from the PMFS to have peak areas over five times their own intercepts of linear response (additional file 9). This sum of SMPP minima must have large errors, as it was just comparable to that estimated by the same approach for a mixture of a single candidate biotin derivative (Table 1).

**Figure 7:**
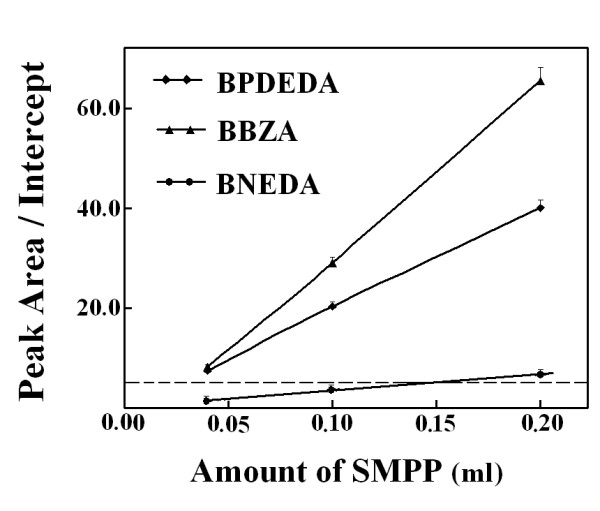
**Effects of SMPP quantities on ratios of the peak areas of each biotin derivative to the absolute value of its intercept of linear response with a PMFS of a mixture of BNEDA and BBZA prepared *via *SPSAG using NEDA and BZA at 1:6 ratio**. Final BPDEDA was 2.0 μM and the expected total concentration of the two candidate biotin derivatives was 4.6 μM.

**Figure 8:**
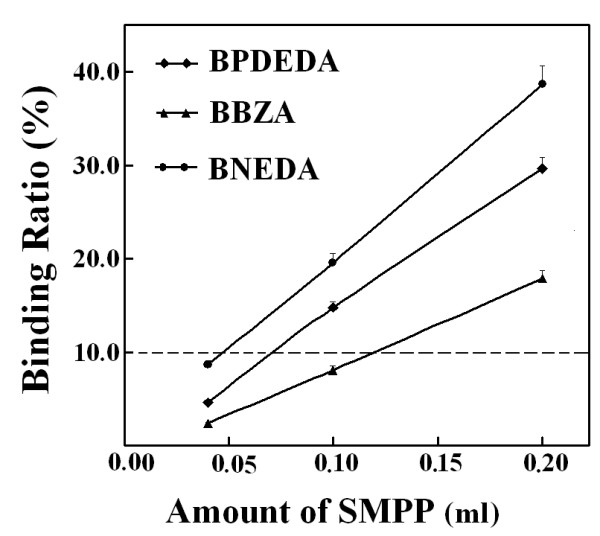
**Effects of SMPP quantities on the binding ratios of each biotin derivative from the PMFS of the mixture of BNEDA and BBZA prepared *via *SPSAG using NEDA and BZA at 1:6 ratio**. Conditions were completely the same as those described in Figure 7.

**Figure 9:**
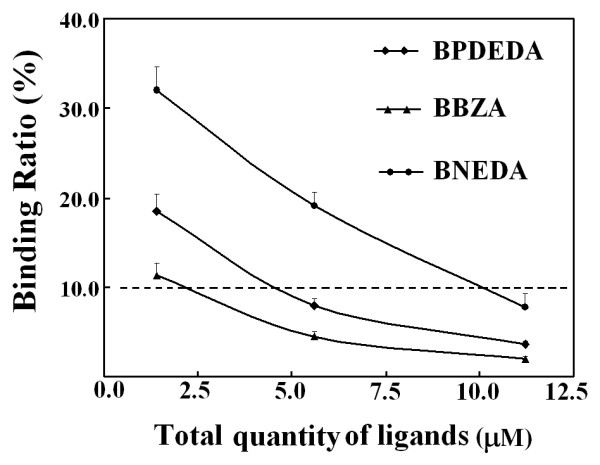
**Effects of the quantities of a PMFS on the ratios of peak areas of each biotin derivative to the absolute value of its intercept of linear response**. The PMFS was that used in Figure 7. SMPP of 0.20 mL was used in the competitive binding systems.

**Figure 10:**
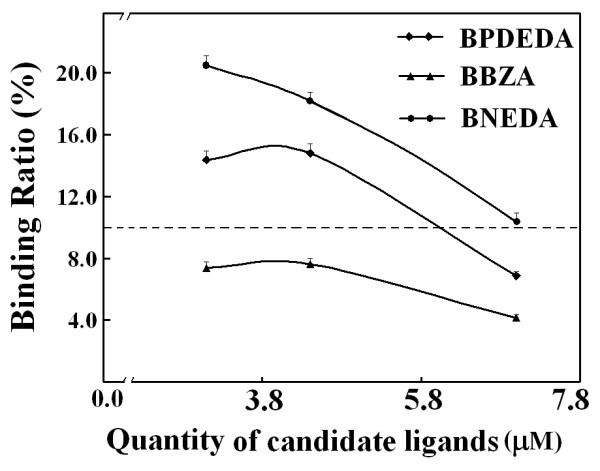
**Effects of the composition ratios of the PMFSs on the ratios of peak areas of each biotin derivative to the absolute value of its intercept of linear response**. The quantity of SMPP was 0.20 mL. The composition ratios of the PMFSs were adjusted by keeping the final BPDEDA at 2.0 μM in the competitive binding systems.

With a representative mixture, an optimized quantity of SMPP was then estimated by the experimental approach and meanwhile optimized PMFS composition ratios were sought. Among the designed biotin derivatives, BNEDA had the highest affinity while BBZA had the lowest affinity (Table 3). Two mixtures of BNEDA and BBZA were thus prepared *via *SPSAG using the two mixtures of BZA and NEDA at molar ratios of 1:6 and 6:1, respectively, and were used as the representative mixtures. The following results were obtained with these two representatives. (a) With a PMFS of the mixture *via *SPSAG using NEDA and BZA at 1:6, the expected total concentration of all candidate biotin derivatives was firstly preset at about 4.6 μM and the final BPDEDA concentration was preset at 2.0 μM in competitive binding systems. In this case, peak areas for each bound biotin derivative in the concentrated extracts (Figure 7), and binding ratios of each biotin derivative (Figure 8), responded linearly to the quantities of SMPP used. As predicted from these responses, Eq.(6) was validated for BBZA and BPDEDA with a minimum quantity of SMPP of just 0.05 mL, but was validated for BNEDA and BPDEDA with a minimum quantity of SMPP of over 0.15 mL. (b) In competitive binding systems containing 0.20 mL SMPP, the binding ratios of BBZA and BNEDA were simultaneously reduced to below 10% when the expected total concentration of the three biotin derivatives was increased to over 11.0 μM (Figure 9). However, when the final BPDEDA concentration in competitive binding systems was fixed at 2.0 μM, the binding ratios of all the biotin derivatives were reduced to below 10% when the expected total concentration of the biotin derivatives was slightly over 8.0 μM (Figure 10). Thus, the optimization of PMFS composition ratios, rather the quantities of a PMFS alone, was more efficient to validate Eq.(6). (c) A PMFS of the other mixture prepared using NEDA and BZA at 6:1 was tested in the competitive binding systems containing 0.20 mL SMPP and BPDEDA at 4.0 μM. Peak areas of BBZA and BPDEDA in the concentrated extracts invalidated Eq.(6) even if the expected total molar concentration of the biotin derivatives was over 40 μM, but the peak areas of BNEDA were always sufficiently high to meet the prerequisites of Eq.(6). If the quantities of SMPP were over 0.60 mL, Eq.(6) was simultaneously validated for BBZA, BNEDA and BPDEDA when the expected total concentration of the candidate biotin derivatives was over 16.0 μM and the final BPDEDA concentration was 4.0 μM. Therefore, for any candidate ligand possessing both a lower affinity and a lower concentration in a mixture sample, there was the need of more time and cost for optimizing conditions to estimate its affinities. (d) Taken together, in each competitive binding system, 0.20 mL SMPP was used, the final BPDEDA concentration was fixed at 2.0 μM and the expected total molar concentration of all candidate biotin derivatives was over 4.6 μM from the first PMFS of any mixture sample. This practical quantity of SMPP was just slightly higher than that estimated by the experimental approach with a PMFS of a representative mixture, but was over sixteen times that estimated by the parameter-dependent approach with Mixture A *via *the pooling of individual compounds (additional file 9). The total molar quantity of the biotin derivatives from the first PMFS was nearly thirty times the molar quantity of SMPP used, and may be universally applied to mixture samples.

**Table 3:** Relative affinities of multiple candidate biotin derivatives in mixture samples prepared *via *the pooling of individual compounds and SPASG.

Mixtures	Compound	BDEDA	BBZA	BCHA	BNEDA	*n*
			
	Mixture	*K*_X_/*K*_A_	*K*_X_/*K*_A_	*K*_X_/*K*_A_	*K*_X_/*K*_A_	
Pooling individual compounds	Mixture A	1.4 ± 0.2	0.6 ± 0.2	1.4 ± 0.2	2.0 ± 0.5	4
	Mixture B or C	1.3 ± 0.2	0.7 ± 0.3	1.4 ± 0.4	2.1 ± 0.6	6
Combinatorial syntheses	Mixture A	1.1 ± 0.3	0.6 ± 0.3	1.4 ± 0.5	2.1 ± 0.6	4
	Mixture B or C	1.1 ± 0.2	0.5 ± 0.1	1.2 ± 0.3	1.9 ± 0.4	6
Homogenous methods	Pure compound	1.3 ± 0.1	0.6 ± 0.1	1.3 ± 0.1	2.0 ± 0.2	2

#### Estimation of affinities of multiple ligands in mixtures via the pooling of individual compounds

Under the aforementioned optimized conditions, the binding ratios of DNAM and DDEDA from the PMFS of Mixture A *via *the pooling of individual compounds were usually below one-eighth that of BBZA in the competitive binding systems. In the presence of 40 μM biotin in a competitive binding system, the binding ratio of each biotin derivative from the PMFS of Mixture A was reduced by over 40%, whereas those of DNAM and DDEDA showed negligible changes (additional file 8). To the denatured SMPP, all the tested biotin derivatives showed undetectable binding, whereas DNAM and DDEDA showed unchanged binding. Therefore, DNAM and DDEDA in Mixture A are weak nonspecific ligands.

Using the aforementioned optimized conditions, Eq.(6) was validated for each biotin derivative with the PMFS of Mixture A *via *the pooling of individual compounds. No outliers were found in the relative affinity of each candidate biotin derivative from Mixture A[34], and the affinity ranking was BNEDA > BCHA ≈ BDEDA > BBZA. The relative affinities of these biotin derivatives were consistent with those estimated by a homogenous method using their purified counterparts as samples (Table 3)[32]. With the PMFSs of Mixtures B and C, Eq.(6) was validated under the optimized conditions. The relative affinity of each candidate biotin derivative in Mixtures B and C had no outliers and was consistent with that in Mixture A. Consistent relative affinities for the two candidate biotin derivatives in Mixtures B and C *via *the pooling of individual compounds were obtained even when their molar ratios were 1:6 or 6:1. There were consistent results when an Agilent 1100 LC-MS system was used to analyze the same mixtures, or when the quantification sensitivity of the HPLC-MS system in use was reduced by 90% (data not given); these results supported the robustness of this method.

#### Estimation of affinities of multiple ligands in mixtures via SPSAG

**Figure 11:**
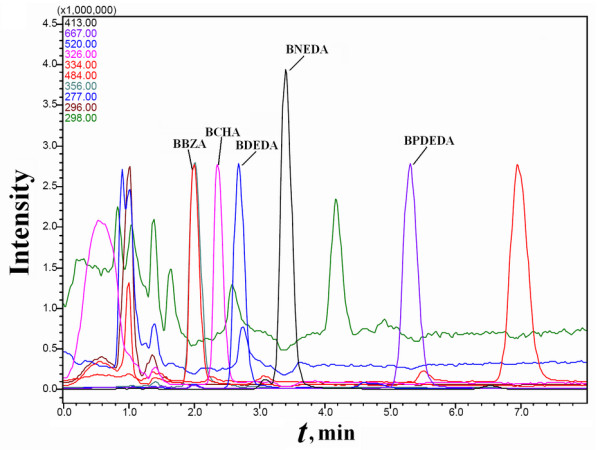
**HPLC-MS-SIM analyses of a PMFS of Mixture A prepared *via *SPSAG**. Only some typical components were monitored during HPLC-MS-SIM analyses. The component with *m*/*z *= 356 around 7.0 min was a contaminant in SMPP, and those with *m*/*z *= 290 around 4.0 min and *m*/*z *= 484 around 7.0 min were expected nonspecific components (additional file 5).

The five biotin derivatives in the PMFS from Mixture A prepared *via *SPSAG were reliably quantified by HPLC-MS-SIM, meanwhile only some of the expected non-biotin derivatives were quantifiable under the same conditions (Figure 11). With the PMFS of Mixture A prepared *via *SPSAG, peak areas of each detected component responded linearly to the quantities of the PMFS with the intercept slightly over zero. Mixture A had approximately equal concentrations of the four candidate biotin derivatives and the total content of the biotin derivatives by mass weight accounted for over 80% of the mixture. The molar ratios of BDEDA and BBZA in Mixture B were found to be about 3.0:1 and 1:3.8 while the molar ratios of BNEDA and BCHA in Mixture C were found to be about 2.6:1 and 1:3.6. In Mixtures B and C, the total content of the biotin derivatives by mass weight was slightly over 60% of the mixture.

**Figure 12:**
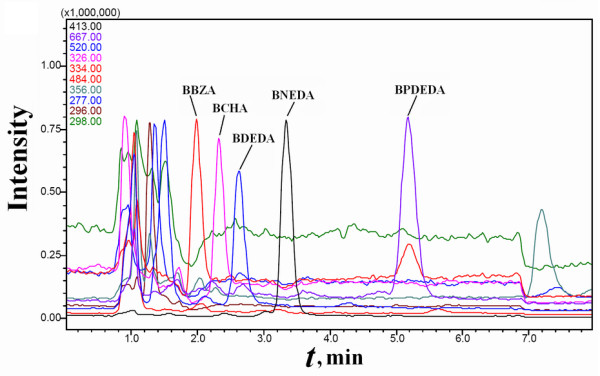
**HPLC-MS-SIM analyses of the concentrated extract from the PMFS of Mixture A prepared *via *SPSAG**. The PMFS was exactly that used in Figure 11.

The average of recovery ratios of all the quantifiable non-biotin components in the PMFS from Mixture A *via *SPSAG was over 30%, with CVs of about 25% (*n *= 3). Among the quantifiable components, only the expected biotin derivatives showed the obvious binding to SMPP (Figure 12). In the presence of 40 μM biotin in a competitive binding system containing the PMFS of Mixture A *via *SPSAG, the binding ratios of the five biotin derivatives were all reduced by over 35%. No components in this PMFS showed detectable binding to SMPP denatured by heating in methanol. Therefore, quantifiable components other than biotin derivatives in the mixtures prepared *via *SPSAG were also not ligands of SMPP.

With the PMFS of Mixture A *via *SPSAG under the optimized conditions, Eq.(6) was validated for each candidate biotin derivative. The relative affinity for each of the four candidate biotin derivatives had no outliers but had the desirable consistency with that from Mixture A *via *the pooling of individual compounds. With Mixtures B and C, the consistent relative affinity for each of the four candidate biotin derivatives was also obtained (Table 3).

## Discussion

To practice this new method with mixture samples, its feasibility and reliability, its overall cost and efficiency, are primarily concerned. Therefore, as for candidate ligands in such mixture samples, their numbers and the differences in their molar quantities should be controlled within reasonable ranges; the experimental conditions should be carefully optimized.

For the feasibility of this new method, a pair of a reference ligand and a candidate ligand should be concurrently quantified in both a PMFS and its concentrated extract by a chromatographic system of high resolution, such as HPLC-MS[35,36]. For any mixture sample of multiple candidate ligands, a preferable reference ligand should have an affinity close to the medium of the affinities of all candidate ligands in the mixture sample so that it can be simultaneously quantified with as many candidate ligands as possible in the concentrated extracts. It is laborious to optimize experimental conditions till Eq.(6) is validated for each pair of a quantifiable candidate ligand from a mixture sample and a suitable reference ligand, when candidate ligands in such a mixture sample have a large number or large differences in their quantities. Hence, this new method is practical for a mixture sample containing a limited number of candidate ligands whose molar quantities have moderate differences, and any candidate ligand whose affinity is lower than a quantified one of any other candidate ligand in the same mixture sample can be abandoned. In practice, a mixture of a single candidate ligand or a racemic mixture of a few pairs of enantiomers can be prepared *via *PCS without purification as a sample for screening by this new method, as long as the overall cost and efficiency are favorable.

For the reliability of this method, all the prerequisites of Eq.(6) should be concomitantly met for a pair of a quantifiable candidate ligand and its reference ligand. In appearance, the concentration ratio, the quantity of the target and that of a PMFS used in competitive binding systems, can be as large as possible to facilitate validating Eq.(6). However, competitive binding among ligands of interest from any PMFS and nonspecific binding of ligands of interest at higher concentrations require the optimizations of both the composition ratio of the PMFS and the quantity of the PMFS in the competitive binding system. The cost of the target also requires the optimization of the quantity of the target used. At higher concentration ratios, the potential interference from contaminants in the target with chromatographic analyses of ligands of interest becomes more significant and there may be precipitates of some ligands of interest from the extract. In fact, the maximum concentration ratios are primarily restricted by the quantities of contaminants of interference from the target, and thus indirectly determined by the quantity of the target used, after the optimization of the solvent(s) to enhance the solubility of ligands of interest. Hence, the composition ratio of ligands of interest in a PMFS, the quantity of the PMFS and that of the target used in competitive binding systems, all should be carefully optimized.

To estimate an optimized quantity of the target, the parameter-dependent approach is simple because it only requires the individual chromatographic parameters of linear response and the individual recovery ratios of all ligands of interest from a PMFS, besides some common parameters (additional file 1). But this approach suffers from large uncertainty due to error propagation, which is more pronounced with mixtures of multiple candidate ligands (additional file 9). In the experimental approach, a representative mixture sample possessing candidate ligands with moderate differences in both their quantities and affinities is needed, and the practical quantity of the target can be slightly higher than an optimized one to enhance universal applicability to different mixture samples. Hence, the experimental approach is preferable, and this assertion was supported by experimental results.

Due to the unknown quantity(ies) of candidate ligand(s) in each mixture sample, PMFS composition ratios have to be carefully optimized. For favorable efficiency in the optimization of PMFS composition ratios, the concentration of a reference ligand (or the content of a mixture sample) in the PMFSs, together with the quantities of the PMFSs in the competitive binding systems, can be fixed so that only the contents of the mixture (or the concentrations of the reference ligand) in the PMFSs need to be optimized. The fine optimization way relies on stepwise adjustment of PMFS composition ratios, and the appearance of stable relative affinities of a candidate ligand in each mixture sample is required. Therefore, the fine optimization way suffers from the unfavorable cost and time involved with seeking optimized PMFS composition ratios for different mixture samples. On the other hand, the rough optimization way adjusts PMFS composition ratios exponentially (Table 2 and 3), and requires the range and intercept of linear response of each ligand of interest to judge the validity of Eq.(6). These required parameters can be easily obtained by chromatographic analyses of a PMFS, as supported by experimentation. More importantly, optimized composition ratios of the first PMFSs can be applied to different mixture samples. Hence, the rough optimization way is preferable.

Under optimized conditions, two candidate biotin derivatives whose affinities were different by 50% were discriminated (Table 2 and 3). Large systematic errors in quantification sensitivity impacted negatively on neither the reliability of ligand affinities nor the universal applicability of optimized conditions. In Eq.(6), only ,  and  are required. There is a close covariance among the quantities of components by chromatographic analyses of a PMFS or its concentrated extract, and thus the propagation of errors from peak areas and recovery ratios into the relative affinity of a candidate ligand is greatly reduced[34]. Hence, the use of a PMFS of each mixture sample accounts primarily for the robustness of this method.

## Conclusions

Taken together, the following conclusions can be drawn.

(1) This method is effective and robust for mixture samples possessing a limited number of candidate ligands whose molar quantities have moderate differences.

(2) This method is effective for candidate ligands of high affinities in mixture samples, as evidenced by the tested biotin derivatives with dissociation constants below 200 fM[31,32].

(3) This method has favorable cost and efficiency for screening candidate ligands of unknown quantity in mixture samples. It only requires standard chromatographic systems and the optimized conditions are generally applicable to most mixture samples from a library.

(4) The integration of PCS with this new method is practical to realize the mixture-based combinatorial library strategy with the desirable overall efficiency and cost.

## Methods

### Materials and chemicals

Streptavidin and SMPP, streptavidin MagneSphere™ paramagnetic particles, were from Promega. *N*-(1-naphthylene)-ethylenediamine (NEDA), cyclohexylamine (CHA), *N*-hydroxysuccinimide (NHS), ethylenediamine (EDA), dicyclohexylcarbodiimide (DCC), diethanolamine (DETA), dansyl chloride (DNS-Cl), morpholine (MPL), and *N*-*t*-Boc-*L*-phenylalanine were from Alfa Aesar. *D*-Biotin (purity > 99%) was from BIO BASIC INC (USA). Benzylamine (BZA) and all other chemicals were reagents of analytic grade or above, and solvents were re-stilled under reduced pressure before use.

### Instruments

Captivate™ magnetic separator (C24703) was from Molecular Probes. A Shimadzu 2010 liquid chromatography system equipped with a 5 μL injector and a 2010A mass spectrometer detector was used. Other instruments were those we used before[31,32].

### Syntheses of individual biotin derivatives

*N*-dansyl-ethylenediamine (DEDA) was prepared as before[32]. *N*-phenylalaninyl-*N*'-dansyl-ethylenediamine (PDEDA) was prepared by condensation of DEDA and *N*-*t*-Boc-phenylalanine, and was purified *via *chromatography after deprotection. NHS-Biotin was prepared as before[31]. Reaction of NHS-Biotin with PDEDA in excess in dichloromethane at room temperature in dark for 24 h yielded BPDEDA that was confirmed by NMR and high-resolution mass-spectrometry. Other biotin derivatives were prepared as before[31,32].

### Mixture of a single candidate ligand prepared *via *PCS

Each mixture of purified NHS-Biotin plus CHA or DETA (1:1.1) in chloroform was kept at room temperature for 6 h. Meanwhile, NHS-Biotin was kept in methanol for 18 h at room temperature. Then, ethanolamine in large molar excess to NHS-Biotin was added to each mixture for reaction of 3 h at room temperature to remove residual NHS-Biotin *via *the production of N-biotinyl ethanolamine. After the removal of solvents at 40°C, biotin derivatives in each mixture were precipitated and washed with ethyl ether. The dried residual served as a mixture sample of a single candidate ligand. The mass content of each candidate biotin derivative in its mixture sample was arbitrarily taken as 50%, unless otherwise stated.

### Mixture of multiple candidate ligands prepared *via *the pooling of individual compounds

DEDA was further dansylated in chloroform to give DDEDA that was roughly purified by repetitive wash with 0.1% HCl and water. DNAM was prepared with bis-(4-aniline)-methane and acetyl anhydride in dichloromethane, followed by repetitive wash with 1% NaOH, 1% HCl and then water. DNAM and DDEDA were pre-mixed at equal molar concentrations, and were further mixed with the indicated candidate biotin derivatives to prepare Mixture A, B and C in methanol, for the average of molar quantities of biotin derivatives equal to that of DDEDA.

### Mixture of multiple candidate ligands prepared *via *SPSAG

2-Hydroxyethyl benzoate was prepared with ethylene glycol and benzoyl anhydride (50:1) in chloroform, followed by repetitive wash with 1% NaOH and water. 2-Hydroxyethyl benzoate, BZA, cyclopentanol and benzylalcohol were then mixed at approximately equal molar concentrations in chloroform, to which was added succinic anhydride (1.05 molar quantity of nuleophiles in total, reflux for 6 h). Then, NHS (1.05 molar quantity of succinic anhydride) and DCC were added (reflux for 12 h) to activate carboxylic acids. After the removal of the precipitates and most solvents, reactive esters in the solution were titrated with NEDA (detected by TLC), and were mixed with NHS-Biotin to make a new mixture of reactive esters (the molar quantity of NHS-Biotin was about one quarter of the total molar quantity of other reactive esters). Meanwhile, three mixtures of the required alkyl primary amines were prepared *via *the pooling of individual compounds, and were used in slight excess to react with the aforementioned new mixture of reactive esters in chloroform to produce Mixtures A, B and C, respectively (Figure 2). Solvents in each reaction mixture were removed under reduced pressure to give a residual that served as a mixture sample after the wash with ethyl ether (additional file 5). Other mixtures were prepared similarly. It was assumed that the total mass content of biotin derivatives was 40% of each mixture and the molar quantity of each biotin derivative was proportional to the molar quantity of the corresponding alkyl primary amine.

### Affinity estimation by this new method

Tris-HCl buffer (0.03 M, pH 7.0) was used throughout. Each PMFS was made by mixing a mixture sample at an indicated quantity with its exogenous reference ligand (BMPL or BPDEDA) at a fixed quantity, both in methanol, to have a final reference ligand at 200 μM in each PMFS. With any mixture of a single candidate ligand, each competitive binding system in 2.0 mL contained 50 μL SMPP (washed with the buffer) and a diluted PMFS at 40 μL. With any mixture of multiple candidate ligands, each competitive binding system in 4.0 mL contained 200 μL SMPP and a PMFS at 40 μL. With a mixture of multiple candidate biotin derivatives, the (expected) total molar quantity of candidate biotin derivatives in competitive binding systems was over 4.6 μM, unless stated otherwise. Each competitive binding system was kept at 25°C for 25 min with gentle shaking at 5-s intervals. Then, target-ligand complexes were recovered *via *magnetic force to extract bound ligands with 1.0 mL methanol at 45°C for 30 min. Each extract was filtered through 0.22 μm membrane; solvents were removed at 40°C to give residuals that were dissolved in 40 μL methanol to serve as the concentrated extract. Each PMFS and its concentrated extract were analyzed, in duplicate, by HPLC-MS-SIM under identical conditions. SMPP were heated in methanol at 50°C for 50 min to detect potential nonspecific binding.

### HPLC-MS-SIM analyses

Phenomenex Gemini 3 μ C_18 _reversed-phase column (10.0 cm × 0.2 cm) was used, and column temperature was maintained at 25°C. The mobile phase was 55% methanol plus 45% aqueous acetic acid (0.3% acetic acid in water) and was used at 0.20 mL per min. M+H^+ ^of each compound upon ESI was monitored. The standard nebulized ESI was carried out with the capillary at 3.5 kV, detector at 1.4 kV and curve desolvation line temperature at 250°C. Each time, 5.0 μL solution was injected for analysis. Each biotin derivative in mixture was identified from its *m*/*z *for M+H^+^, retention time, isotope abundance and co-chromatography with its purified counterpart. Each PMFS and its extract of bound ligands were analyzed consecutively.

### Determination of recovery ratio

A mixture of a purified candidate biotin derivative and its reference ligand (BMPL or BPDEDA) at equal molar quantities was added to Tris-HCl buffer in 2.0 mL containing 400 μL SMPP for the total binding capacity of over 1.6 nmol, and each biotin derivative of interest had the final level of 0.2 μM. Other operations were the same as those for affinity estimation and concentrations of biotin derivatives were determined by HPLC-MS-SIM. Peak areas of each biotin derivative of interest, in both the PMFS and its concentrated extract of bound ligand, were kept over five times the absolute value of its intercept of linear response but within its linear range by proper dilution. Recovery ratio was the percentage of the peak area of a biotin derivative in its concentrated extract to that in the binding mixture minus SMPP after the concentration effect was corrected. Relative recovery ratio was the percentage of the recovery ratio of a candidate biotin derivative to its reference ligand. Recovery ratio of a non-biotin component was its conservation percentage after being heated at 45°C in methanol for 30 min.

### Homogenous competitive assay of affinity

With tryptophan residues in streptavidin as intrinsic donors while fluorescent biotin derivatives as the förster-resonance-energy-transfer acceptor, the homogenous competitive assay of affinity was realized by monitoring streptavidin fluorescence at 340 nm as before[32].

### Data processing and statistic analyses

Relative affinity was calculated from peak areas *via *Eq.(6). Data were the mean ± standard deviation. Student's *t*-test was used to compare differences with *P *< 0.05 as the confidence limit.

## Abbreviations

BBZA: *N*-biotinyl-benzylamine; BCHA: *N*-biotinyl-cyclohexylamine; BDEDA: *N*-biotinyl-*N*'- dansyl-ethylenediamine; BDETA: *N*-biotinyl-diethanolamine; BME: Methyl biotin ester; BMPL: *N*-biotinyl morpholine; BNEDA: *N*-biotinyl-*N*'-(1-naphthyl)-diethyleneamine; BPDEDA: *N*-(*N*_α_-biotinyl-phenylalaninyl)-*N*'-dansyl-ethylenediamine; CV: coefficient of variation; DCC: dicyclohexylcarbodiimide; HPLC-MS: high-performance-liquid-chromatography mass spectrometry; HPLC-MS-SIM: HPLC-MS *via *selective-ion-monitor; HTS: high-throughput-screening; NHS-Biotin: biotinyl N-hydroxylsucciniimide ester; PCS: parallel combinatorial synthesis; PMFS: processed-mixture-for-screening; SMPP: streptavidin MagneSphere™ paramagnetic particles from Promega; SPSAG: solution-phase simultaneous addition of groups;

## Authors' contributions

XLY took part in conceiving the idea, interpreted structures of most compounds and wrote most parts of the manuscript; YLX did most parts of experiments and processed most chromatographic data; JP and HZ did some experiments and took part in writing the manuscript; YWC designed some routes of syntheses and interpreted structures of some compounds; JL, GBL, CZ, YHY, SZ, HDY, took part in conceiving the idea, did parts of experiments, took part in writing the manuscript; FL conceived the idea, wrote both the project proposal and the whole manuscript, and led the group. All authors read and approved the final manuscript.

## Supplementary Material

Additional file 1 **the parameter-dependent approach to an optimized quantity of target**. Click here for file

Additional file 2 **two ways to optimize PMFS composition ratio**. Click here for file

Additional file 3 **structures of biotin derivatives used in this work**. Click here for file

Additional file 4 **Interpretation of structure of BPDEDA**. Click here for file

Additional file 5 **expected ions upon ESI for compounds *via *SPSAG**. Click here for file

Additional file 6 **linear response for HPLC-MS-SIM analyses of a biotin derivative used with each mixture of a single candidate ligand**. Click here for file

Additional file 7 **linear response for HPLC-MS-SIM analyses of a biotin derivative used with each mixture of multiple candidate ligands**. Click here for file

Additional file 8 **screening of Mixture A prepared *via *the pooling of individual compounds**. Click here for file

Additional file 9 **parameters for HPLC-MS-SIM analyses of a PMFS of Mixture A and the parameter-dependent approach to an optimized quantity of SMPP**. Click here for file
