# Supplementary material for: Estimation of affinities of ligands in mixtures via magnetic recovery of target-ligand complexes and chromatographic analyses: chemometrics and an experimental model
Source: BMC Biotechnol. 2011 May 5;11:44. doi: 10.1186/1472-6750-11-44 (PMC3096923; doi:10.1186/1472-6750-11-44)
Supplement: Additional file 1 — the parameter-dependent approach to an optimized quantity of target. [file 1472-6750-11-44-S1.PDF]

The parameter-dependent approach to an optimized quantity of target

With any mixture sample, the minimum quantity of a target in a competitive binding system could be approximated step-by-step with some parameters as described below.

(a) From the slope and intercept of linear response, the volume of solutions for analysis, the minimum concentration of each ligand of interest in the concentrated extract to validate Eq.[6] ($C_{x1,i}$) is derived.

(b) The total molar quantity of such a ligand of interest in the concentrated extract (N_{x1}) is equal to $N_{x1,i}= C_{x1,i} \times V_0$.

(c) The minimum quantity of each ligand of interest bound by the target in the competitive binding system ($N_{x2,i}$) could be derived from its recovery ratio. This total molar quantity of such a ligand of interest bound by the target in the competitive binding system is $N_{x2,i} = C_{x1,i} \times V_0/RR_{X,i}$

(d) The minimum quantity of the target in a competitive binding system to bind the minimum quantity of each ligand of interest ($N_{x3,i}$) was derived from the specific binding capacity of the target (N_{sb}). That is, $N_{x3,i} = N_{x2,i} / N_{sb} =C_{x1} \times V_0/RR_{X,i}/ N_{sb}$.

(e) The sum of the minimum quantities of the target for all ligands of interest from a PMFS gave the minimum quantity of the target in the competitive binding system as N_{tt}, and $N_{tt} = \sum N_{x3,i}$

Such an approximation process was demonstrated in Table 1 and Table S2. Any practical quantity of the target in competitive binding systems should be much larger than such a sum so that there can be reasonable fluctuation of bound ligands in the extract to validate Eq. (6).
