# Supplementary material for: Estimation of affinities of ligands in mixtures via magnetic recovery of target-ligand complexes and chromatographic analyses: chemometrics and an experimental model
Source: BMC Biotechnol. 2011 May 5;11:44. doi: 10.1186/1472-6750-11-44 (PMC3096923; doi:10.1186/1472-6750-11-44)
Supplement: Additional file 2 — two ways to optimize PMFS composition ratio. [file 1472-6750-11-44-S2.PDF]

Two ways to optimize PMFS composition ratios

fine optimization of PMFS composition ratios

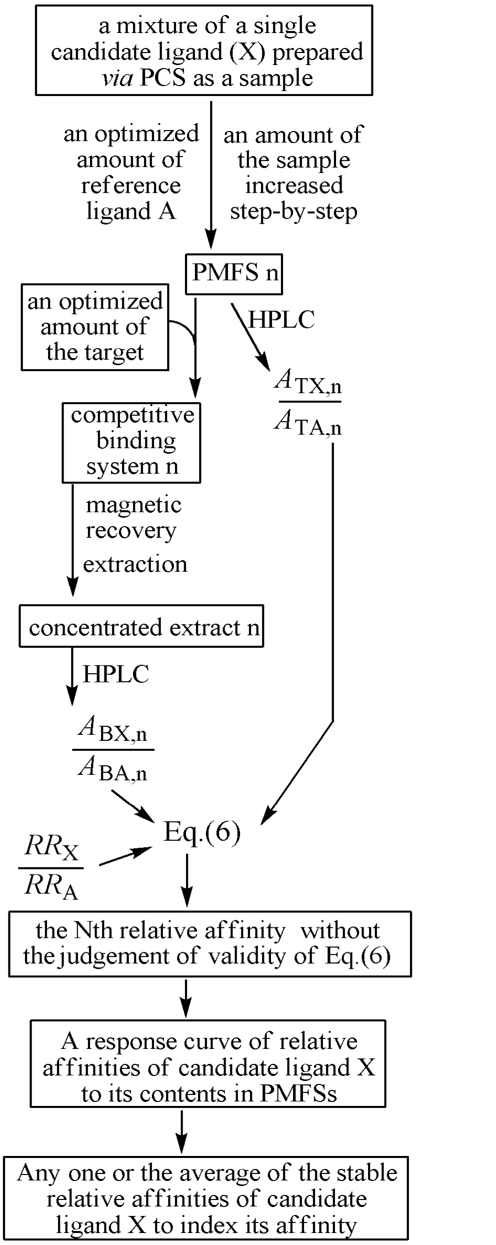

$\frac{A_{BX,\,n}}{A_{BA,\,n}}$ The Nth ratio of the amount of the bound candidate ligand X to that of the bound reference ligand with PMFS n

The amount of reference ligand A, as well as that of the target protein, was fixed. See text part for Eq.(6) to derive the relative affinity

rough optimization of PMFS composition ratios

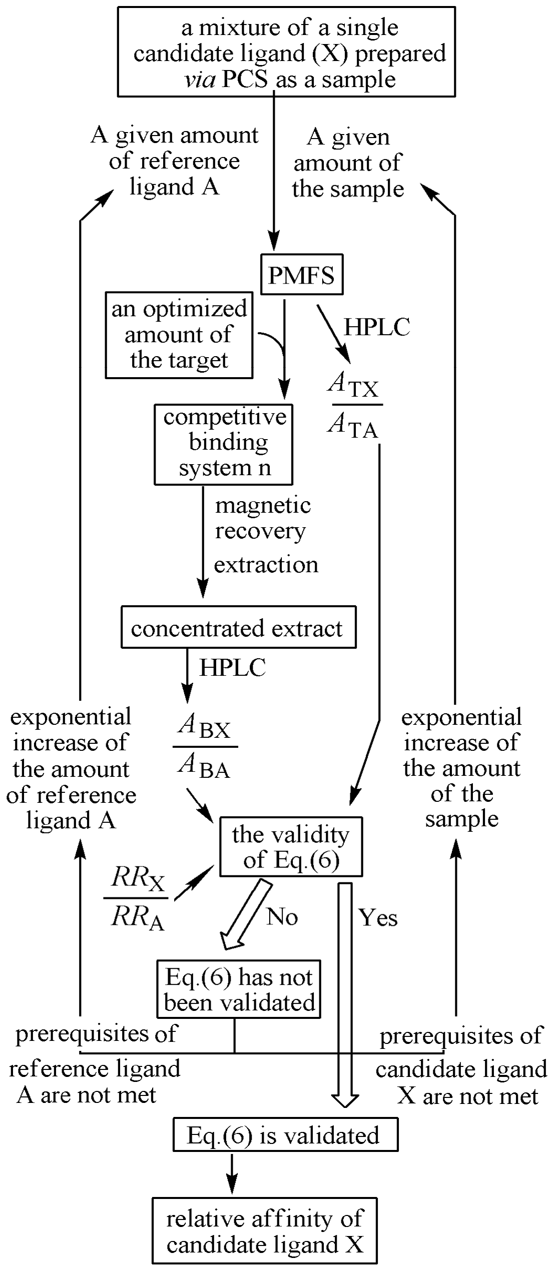

$\frac{A_{TX,\,n}}{A_{TA,\,n}}$ The Nth ratio of the total amount of the candidate ligand X to that of the reference ligand with PMFS n

$\frac{RR_X}{RR_A}$ Relative recovery ratio determined independently
