# Supplementary material for: Estimation of affinities of ligands in mixtures via magnetic recovery of target-ligand complexes and chromatographic analyses: chemometrics and an experimental model
Source: BMC Biotechnol. 2011 May 5;11:44. doi: 10.1186/1472-6750-11-44 (PMC3096923; doi:10.1186/1472-6750-11-44)
Supplement: Additional file 3 — structures of biotin derivatives used in this work. [file 1472-6750-11-44-S3.PDF]

Structures of biotin derivatives used in this work

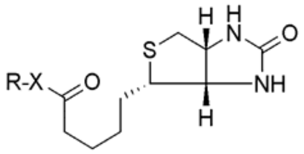

nucleophile (X-H)	name	abbreviation	Calculated m/z$^+$ (+Na^+ or +H^+)	m/z$^+$ by HRMS
H_3C-OH	Methyl Biotin Ester	BME	280.0926 (+Na^+)	280.0931
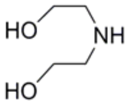	*N*-Biotinyl-diethanolamine	BDETA	354.1469 (+Na^+)	354.1474
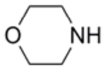	*N*-Biotinyl-morpholine	BMPL	314.1538 (+H^+)	314.1544
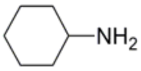	*N*-Biotinyl-cyclohexanylamine	BCHA	326.1902 (+H^+)	326.1900
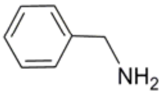	*N*-Biotinyl-benzylamine	BBZA	334.1589 (+H^+)	334.1594
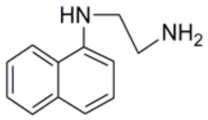	*N*-Biotinyl-*N*'-(1-naphthylene) -ethylenediamine	BNEDA	413.2011 (+H^+)	413.2008
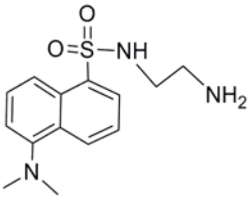	*N*-Biotinyl-*N*'-dansyl -ethylenediamine	BDEDA	520.2052 (+H^+)	520.2047
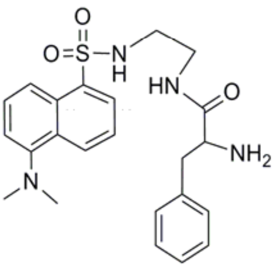	*N*-(*N*α-Biotinyl-phneylalaninyl) -*N*'-dansyl-ethylenediamine	BPDEDA	667.2736 (+H^+)	667.2719
