# Supplementary material for: Estimation of affinities of ligands in mixtures via magnetic recovery of target-ligand complexes and chromatographic analyses: chemometrics and an experimental model
Source: BMC Biotechnol. 2011 May 5;11:44. doi: 10.1186/1472-6750-11-44 (PMC3096923; doi:10.1186/1472-6750-11-44)
Supplement: Additional file 4 — Interpretation of structure of BPDEDA. [file 1472-6750-11-44-S4.PDF]

Interpretation of structure of BPDEDA

EI-MS *m/z*: 667(M+H$^+$). HRMS: calcd for $C_{33}H_{43}N_6O_5S_2$ (M+H$^+$), 667.2736; found, 667.2719.

^{1}HNMR (500 MHz, DMSO, δ) for $C_{33}H_{42}N_6O_5S_2$: δ=1.15(m,2H,C-CH_2-C,biotin-), 1.37(m,2H,C-CH_2-C-CO,biotin-), .37,1.53(m,C-CH_2-C-S,biotin-),2.01(t,2H,J=7.8Hz,C-CH_2-CO-,biotin-),2.55(d,1H,J=12.0Hz,C-CH_2-S-,biotin-),2.86(dd,1H,J=5.3,13.7Hz，C-CH_2-S-,biotin-), 3.05 (m, 1H, C-CH-S-, biotin-), 4.06(m,1H, J=5.0Hz,-CH-CH-, biotin-), 4.27(t,1H,J=5.2Hz,-CH-CH-, biotin-),6.33(s,1H,-NH-CO-N-,biotin-),6.37(s,1H,-H-CO-NH-,biotin-);2.66(m,2H,SO_2-N-CH_2-C-N, EDA-),2.98(m,2H,-CH_2-N-CO,EDA-),7.58(t,1H,J=8.1Hz,SO_2-NH-C,EDA-),7.92(m,1H,-C-NH-CO ,EDA-);.69(m,2H,C-CH_2-C*,phe),4.35(m,1H,CO-CH-N(-C-C*)-,Phe-),7.13(m,3H,Phe-),7.89(m,2H ,-C*H-,Phe-),8.27(s,1H,CO-NH-C-C*(CO)-,Phe-);3.06(s,6H,CH_3-N(C*)-CH_3,DNS-),7.13(m,1H,DNS-),7.23(d,1H,J=7.6Hz,DNS-),7.63(t,1H,J=7.8Hz,DNS-),8.08(d,1H,J=6.6Hz,DNS-),8.26(d,1H,J= 8.6Hz, DNS-),8.46(d,1H,J=8.6Hz,DNS-).

^{13}CNMR (500 MHz, DMSO, δ) for $C_{33}H_{42}N_6O_5S_2$: 172.67(C(=O),1-amide);171.84(C(=O), 1-amide),163.21(C(=O),N-urea,biotin-),61.47(CH,tetrahydrothiophene,biotin-),59.69(CH,tetrahydrothiophene,biotin-),55.85(CH,tetrahydrothiophene,biotin-),42.12(CH_2,tetrahydrothiophene,biotin-), 35.36(CH_2,aliphatic,biotin-),28.40(2CH_2,aliphatic,biotin-),C25.57(CH_2,aliphatic,biotin-);151.87(C, 1-naphthalene),136.21(C,1-naphthalene),129.97(C,1-naphthalene),129.58(CH,1-naphthalene),129.53(CH,1-naphthalene),128.78(CH,1-naphthalene),128.38(CH,1-naphthalene),124.06(CH,1-naphthalene),119.47(C,1-naphthalene),115.64(CH,1-naphthalene);126.58(C,1-benzene),128.38(2CH,1-benzene),129.53(2CH,1-benzene),138.35(C,1-benzene),54.33,(CH,aliphatic,-benzene)38.16(CH_2,aliphatic,-benzene),45.52(2C,aliphatic,DNS-);45.52(CH_2,aliphatic,EDA-),39.04(CH_2,aliphatic,EDA-).
