# Supplementary material for: Estimation of affinities of ligands in mixtures via magnetic recovery of target-ligand complexes and chromatographic analyses: chemometrics and an experimental model
Source: BMC Biotechnol. 2011 May 5;11:44. doi: 10.1186/1472-6750-11-44 (PMC3096923; doi:10.1186/1472-6750-11-44)
Supplement: Additional file 5 — expected ions upon ESI for compounds via SPSAG. [file 1472-6750-11-44-S5.PDF]

Expected ions upon ESI for compounds via SPSAG

Carboxylic acid (activated as NHS esters)	DEDA (293.4)	BZA (107.2)	CHA (99.2)	NEDA (186.0)
Benzoyl-ethyleneglycol-succinate	541.6	355.4	347.4	434.2
Cyclopentanol-succinate	461.6	275.3	267.4	354.2
Benzylamine-succinate	482.6	296.4	288.4	375.2
Benzylalcohol-succintae	483.6	297.3	289.4	376.2
Biotin (NHS-Biotin)	519.4	333.4	325.5	412.3
