# Supplementary material for: Estimation of affinities of ligands in mixtures via magnetic recovery of target-ligand complexes and chromatographic analyses: chemometrics and an experimental model
Source: BMC Biotechnol. 2011 May 5;11:44. doi: 10.1186/1472-6750-11-44 (PMC3096923; doi:10.1186/1472-6750-11-44)

Linear response for HPLC-MS-SIM analyses of a biotin derivative used with each mixture of a single candidate ligand

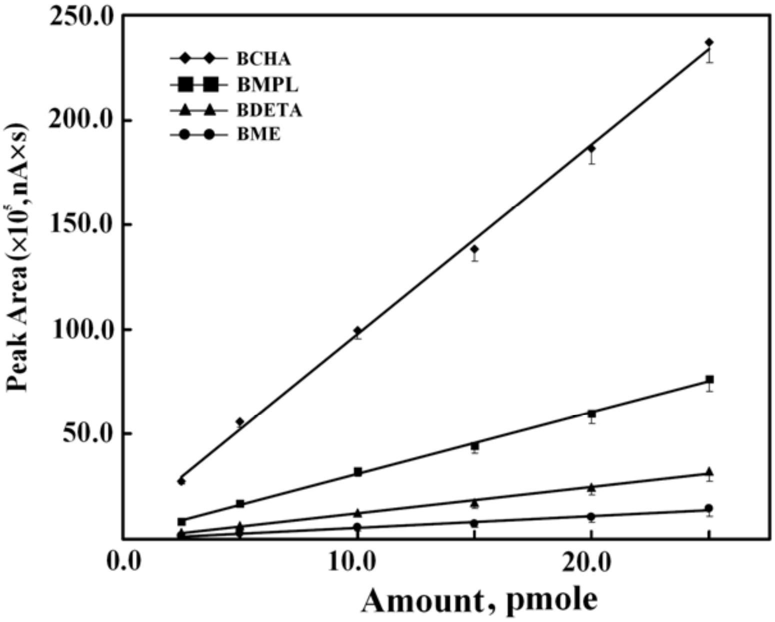

Supplement: Additional file 6 — linear response for HPLC-MS-SIM analyses of a biotin derivative used with each mixture of a single candidate ligand. [file 1472-6750-11-44-S6.PDF]
