# Supplementary material for: Estimation of affinities of ligands in mixtures via magnetic recovery of target-ligand complexes and chromatographic analyses: chemometrics and an experimental model
Source: BMC Biotechnol. 2011 May 5;11:44. doi: 10.1186/1472-6750-11-44 (PMC3096923; doi:10.1186/1472-6750-11-44)
Supplement: Additional file 8 — screening of Mixture A prepared via the pooling of individual compounds. [file 1472-6750-11-44-S8.PDF]

Screening of Mixture A prepared *via* the pooling of individual compounds

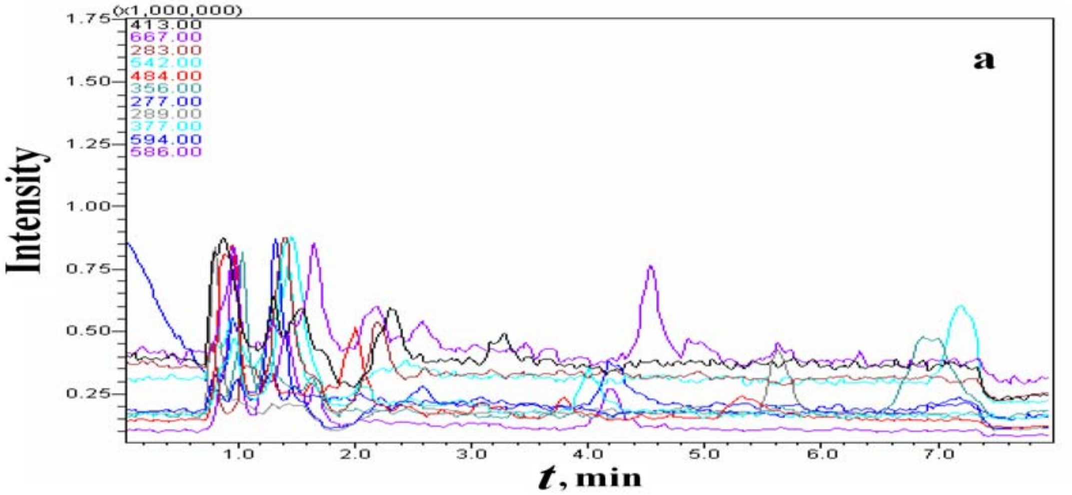

(a) *m/z*	484	586	667	289	356	377
retention time (min)	1.9	4.2	4.6	5.6	7.2	7.4

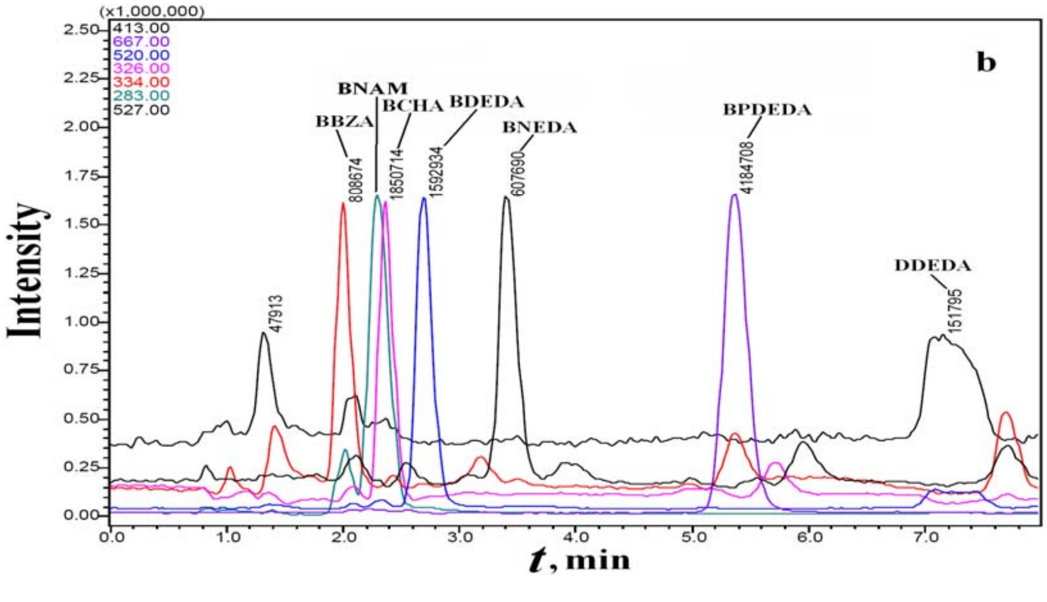

(b) *m/z*	413	667	520	326	334	283	527
retention time (min)	3.5	5.4	2.7	2.4	2.1	2.1	7.2
Peak areas ($\times 10^4$)	60.8	418.5	159.3	185.1	80.9	1083.9	15.2

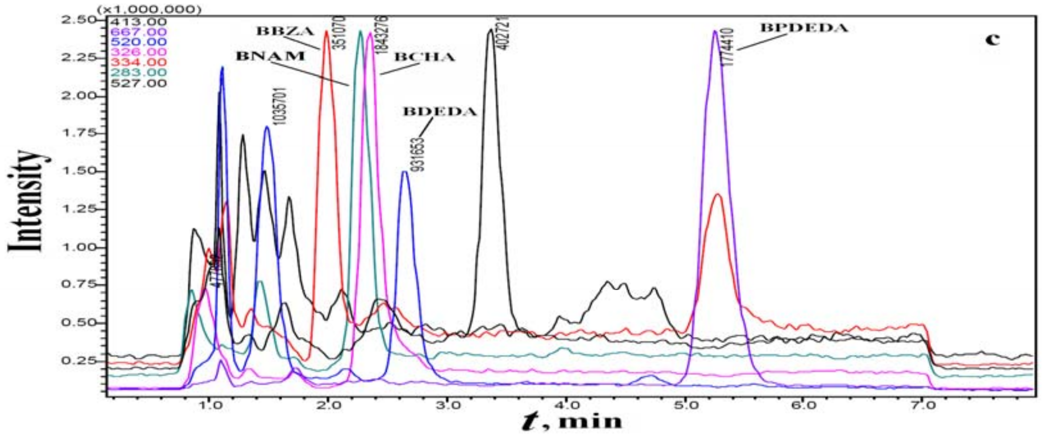

(c)

m/z	413	667	520	326	334	283	527
retention time (min)	3.5	5.4	2.7	2.4	2.1	2.1	7.2
Peak area($\times 10^4$)	40.3	177.4	93.2	184.3	35.1	88.1	undetected

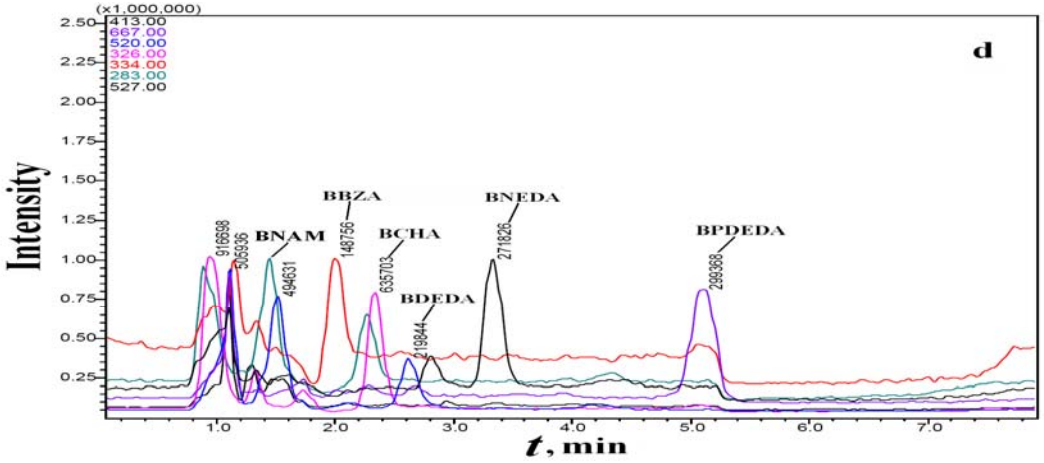

(d)

m/z	413	667	520	326	334	283	527
retention time (min)	3.5	5.4	2.7	2.4	2.1	2.1	7.2
Peak area($\times 10^4$)	27.2	29.9	2.0	63.6	14.9	75.9	undetected

(a) contaminants from 0.10 ml magnetic nanoparticles concentrated into 80 μl extract.

(b) the properly diluted PMFS with Mixture A from pooling individual compounds.

(c) the corresponding concentrated extract (the concentration ratio was 25).

(d) the extract in the presence of 20 μM natural biotin (the concentration ratio was 25).
