# Supplementary material for: Estimation of affinities of ligands in mixtures via magnetic recovery of target-ligand complexes and chromatographic analyses: chemometrics and an experimental model
Source: BMC Biotechnol. 2011 May 5;11:44. doi: 10.1186/1472-6750-11-44 (PMC3096923; doi:10.1186/1472-6750-11-44)
Supplement: Additional file 9 — parameters for HPLC-MS-SIM analyses of a PMFS of Mixture A and the parameter-dependent approach to an optimized quantity of SMPP. [file 1472-6750-11-44-S9.PDF]

Parameters for HPLC-MS-SIM analyses of a PMFS of Mixture A and the parameter-dependent approach to an optimized quantity of SMPP.

compound	BPDEDA	BDEDA	BNEDA	BCHA	BBZA
m/z for $M+H^+$	667	520	413	326	334
slope	13.8	21.6	9.4	25.3	14.2
intercept	1.69	1.82	1.25	1.95	–1.80
Correlation coefficient	0.9998	0.9995	0.9990	0.9994	0.9996
Minimum quantity in 5μl extract to validate Eq.(1) (pmole) [a]	0.61	0.42	0.66	0.39	0.76
Minimum quantity in 40 μl extract to validate Eq.(1)	4.9	3.4	5.3	3.1	6.1
Recovery ratios	0.49	0.46	0.44	0.54	0.43
Minimum quantity that is bound by SMPP (pmole)	10.0	7.3	12.1	5.7	14.5
Minimum quantity of SMPP(μl) [b]	2.5	1.8	3.0	1.4	3.6
Sum of minima with Mixture A (μl)			12.3		

[a] calculated with the binding capacity of 4 nmole per mL SMPP.

[b] calculated with the binding capacity of 4 nmole per mL SMPP.
